# Intensity of insecticide resistance in the major malaria vector *Anopheles funestus* from Chikwawa, rural Southern Malawi

**DOI:** 10.1186/s13071-022-05299-3

**Published:** 2022-06-21

**Authors:** Justin Kumala, Lizette L. Koekemoer, Maureen Coetzee, Themba Mzilahowa

**Affiliations:** 1 Malaria Alert Centre-Communicable Diseases Action Centre (MAC-CDAC), Kamuzu University of Health Sciences, Blantyre, Malawi; 2 Wits Research Institute for Malaria, School of Pathology, Faculty of Health Sciences, University of the Witwatersrand, Johannesburg, South Africa; 3 Centre for Emerging Zoonotic & Parasitic Diseases, National Institute for Communicable Diseases, Johannesburg, South Africa

**Keywords:** Pyrethroids, Susceptibility test, Bioassay, Insecticide-treated nets, Indoor residual spraying, Malaria transmission, Vector control, Sub-Saharan Africa

## Abstract

**Background:**

Malaria vector control using insecticide-based approaches has proven to be an effective strategy. However, widespread insecticide resistance among malaria vector populations across sub-Saharan Africa threatens to derail control efforts. This study was conducted in Chikwawa district, an area in rural southern Malawi characterised by persistent malaria transmission and reports of insecticide resistance in the local mosquito population. The aim of the was to characterise the intensity of insecticide resistance within a population of *Anopheles funestus* sensu lato (s.l.)*,* a major vector of malaria in this district*.*

**Methods:**

Live adult females belonging to the *An. funestus* group were collected from households by indoor aspiration. The CDC bottle assay was used for phenotypic quantification of resistance to deltamethrin, permethrin and alpha-cypermethrin at 1×, 2.5×, 5× and 10× the recommended diagnostic dose for each of these insecticides. WHO tube assays were used to determine susceptibility to bendiocarb, dichlorodiphenyltrichloroethane (DDT) and pirimiphos-methyl insecticides at diagnostic concentrations.

**Results:**

*Anopheles funestus* s.l. exposed to 10× the recommended diagnostic dose was highly resistant to alpha-cypermethrin (mortality 95.4%); in contrast, mortality was 100% when exposed to both deltamethrin and permethrin at the same dose. Despite showing susceptibility to deltamethrin and permethrin at the 10× concentration, mortality at the 5× concentration was 96.7% and 97.1%, respectively, indicating moderate resistance to these two insecticides. WHO susceptibility assays indicated strong resistance against bendiocarb (mortality 33.8%, *n* = 93), whereas there was full susceptibility to DDT (mortality 98.9%, *n* = 103) and pirimiphos-methyl (mortality 100%, *n* = 103).

**Conclusions:**

Strategies for managing resistance to insecticides, particularly against pyrethroids, must be urgently implemented to maintain the effectiveness of insecticide-based vector control interventions in the area. Such strategies include the wide-scale introduction of third-generation synergist insecticide-treated bed nets (ITNs) and next-generation dual active ingredient ITNs. The use of effective non-pyrethroids, such as pirimiphos-methyl, clothianidin and potentially DDT, could provide a window of opportunity for indoor residual spraying across the district. This strategy would support the current Malawi Insecticide Resistance Management Plan which aims at rotating insecticides to minimise selection pressure and slow down the evolution of resistance to approved insecticides. These actions will help to prevent malaria vector control failure and improve progress towards malaria elimination.

**Graphical Abstract:**

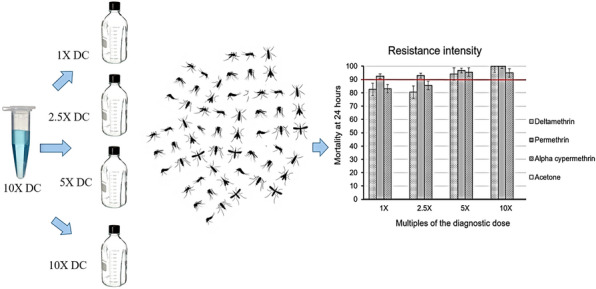

## Background

Good progress had been made in reducing the burden of malaria in the African region over the past two decades, despite stalls in progress and an increase in cases between 2020 and 2021 associated with disruptions in the implementation of control programs due to the COVID-19 pandemic [1]. In the African region, it was estimated that *Plasmodium falciparum* prevalence declined by 50% while incidence decreased by 42% between 2000 and 2019 [2]. In Malawi, the implementation of evidence-based policies, including the provision of indoor residual spraying (IRS), insecticide-treated bed nets (ITNs) and intermittent preventive therapy for pregnant women (iPTp), has contributed to a decline in malaria prevalence from 44% in 2010 to 22% by 2017 [3]. Mean modelled prevalence rates of *P. falciparum* declined from 29% in 2010 to 15% in 2017, with the percent decline ranging from 3 to 79% in some areas [4]. However, these gains are threatened by increases of insecticide resistance in malaria vector populations, both locally and across sub-Saharan Africa [5–9].

In Malawi, resistance to at least one insecticide has been reported in many areas of the country, including resistance to multiple insecticides in Chikwawa district. Chikwawa is an area along the Lower Shire Valley. Malaria transmission is persistent in this area, and it is known to be among the highest transmission risk areas, with *P. falciparum* prevalence rates as high as 15% (*n* = 9646) among participants, as reported in a recent study [10]. *Anopheles funestus* sensu stricto (s.s.), the primary malaria vector in this district, has shown reduced susceptibility to pyrethroids and carbamates, with studies indicating that this resistance is enzyme-mediated [11–13]. *Anopheles arabiensis*, a secondary vector in the district, has also shown reduced levels of susceptibility to pyrethroids [11, 12, 14]. However, the extent to which the rise of insecticide resistance might be hampering malaria control efforts in the district remains under-explored.

The level of pyrethroid resistance in Malawian *An. funestus* sensu lato (s.l.) has slowly risen since it was first reported in 2008 by Hunt et al. [15]. In 2015, reports from Chikwawa district indicated an increase in multiple resistance mechanisms in the local *An. funestus* population, namely over-expression of the pyrethroid resistance genes C*YP6Pa/b* and *CYP7M7* and presence of the dieldrin resistance mutation (A296S-RDL) [13, 16, 17]. Such reports suggest that insecticide-based vector control tools (such as ITNs and IRS) may slowly lose their effectiveness over time, especially in the wake of multiple resistance, as recently reported in neighbouring Mozambique where the same resistance mechanisms in the local *An. funestus* population have induced a loss of efficacy of piperonyl-butoxide (PBO)-based ITNs [18], indicating other uncharacterised mechanisms at play. In Cote d’Ivoire, PBO pre-exposure of resistant *An. gambiae* s.l. did not yield full restoration of susceptibility to pyrethroids and neither was there full susceptibility to chlorfenapyr, a novel insecticide used for public health purposes in combination with pyrethroids in the newer generation ITNs [19].

There is a lack of compelling evidence-based data in Malawi on the nature of the relationship—if any−between insecticide resistance and the effect of ITNs on mosquito mortality in the field. Specifically, it is unknown whether malaria transmission control is compromised in people using ITNs and whether there is any relationship between mosquito resistance and epidemiological profiles of clinical malaria in the district. These factors are important in establishing the real impact of resistance to operational malaria control [20–22]. In this context, the aim of this study focused on determining the intensity of resistance of mosquitoes to deltamethrin, permethrin and alpha-cypermethrin (pyrethroids) and alternative non-pyrethroid insecticides. The study was part of a larger study which aimed at investigating the impact of insecticide resistance on vector control efforts in the district. The main driver of this study was the need to determine the operationally significant level of resistance that may serve as an early warning signal of potential loss of effectiveness in order to guide the local vector control intervention policy and minimise or prevent control failure.

## Methods

### Study design

**Fig. 1:**
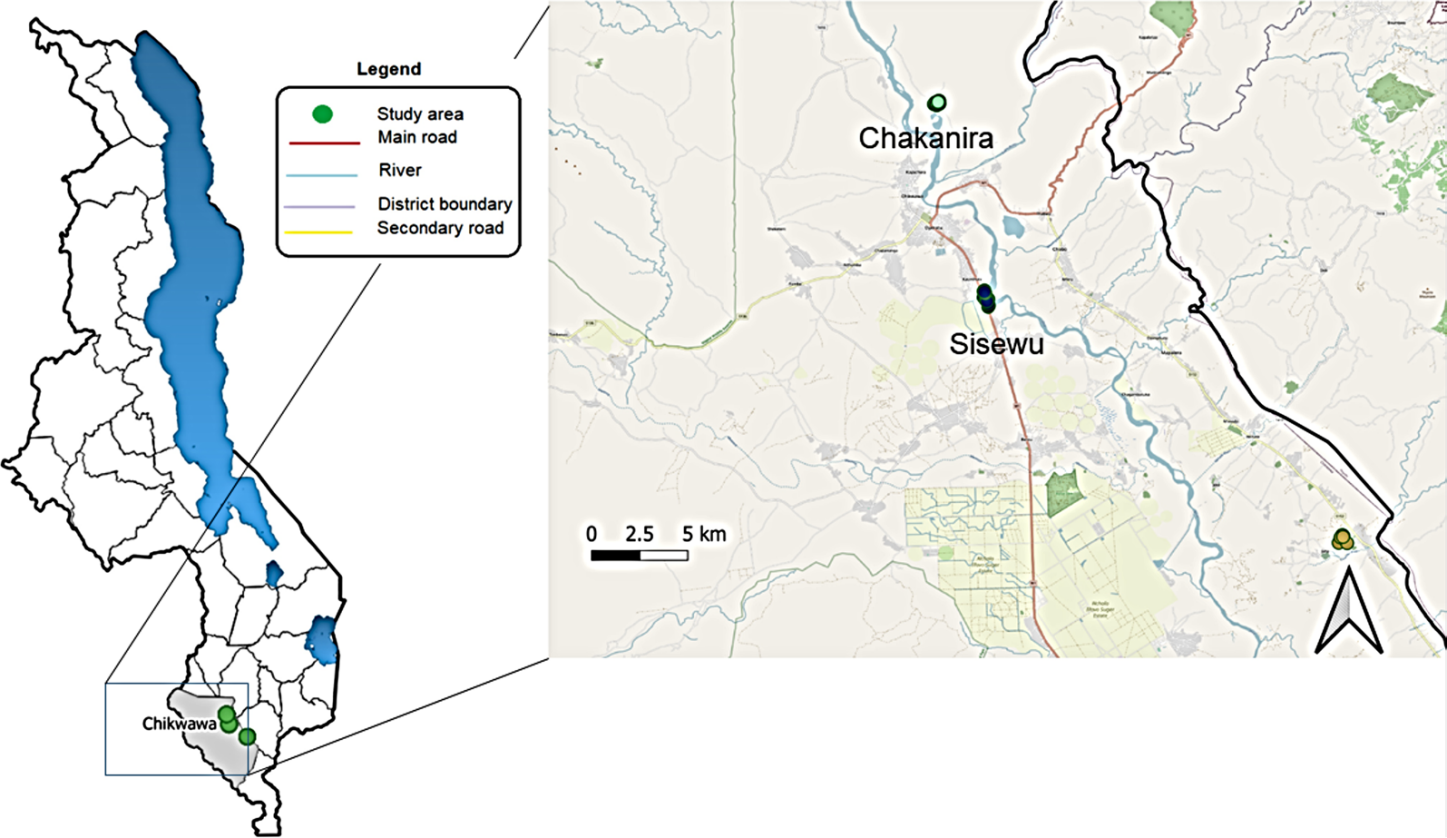
Map of Malawi, showing the location of Chikwawa district and study sites (Source: QGIS 3.18.3)

This entomological study was conducted after the rainy season, between April and July 2018, across two malaria sentinel sites, Chakanira (15°58′54.1″ S, 34°48′09.1″ E) and Sisewu (16°04′39.9″ S, 34°49′39.9″ E) villages in Chikwawa district, southern Malawi (Fig. 1). Malaria transmission is perennial in this area, but cases peak at the height of the rainy season, usually between November and April [23].

### Mosquito collections

Collections of live adult mosquitoes were carried out inside houses using a battery-powered Prokopak aspirator (John W Hock Co., Gainesville, FL, USA)). As this method collects resting mosquitoes, there was a chance that some of the captured mosquitoes would had a blood meal or a partial blood meal. Hence, following capture, all mosquitoes were held for 1–2 days to allow them to rest and also digest any existing blood meals, if any [24], ensuring that the mosquitoes used in bioassays were free from any blood meals. The absence of any blood meal was also verified visually prior to the bioassay. Following capture in the field, mosquitoes were placed in paper cups which were placed in cooler boxes and provided with a sugar solution prior to transport to the laboratory in Blantyre. The local vectors are *An. funestus* s.s. (most abundant) and *An. arabiensis* [25]. All mosquitoes used in this study were wild-caught female *An. funestus* group mosquitoes of unknown age; no specimens were reared in the laboratory or insectary for the insecticide susceptibility tests. The WHO protocol allows for the direct use of wild-caught mosquitoes for bioassays as they are operationally relevant [26].

### CDC bottle assays

Tests were performed on three replicates of 20–25 wild-caught adult females of unknown age. These mosquitoes were exposed for 1 h in 250-ml Wheaton glass bottles coated with a pyrethroid insecticide (permethrin, deltamethrin or alpha-cypermethrin) at a concentration of 1×, 2.5× DC, 5× DC and 10× of the respective diagnostic concentration (DC). Final concentrations were: (i) deltamethrin: DC = 12.5 µg/ml, 2.5× DC = 31.3 µg/ml, 5× DC = 62.5 µg/ml, 10× DC = 125 µg/ml; (ii) permethrin: DC = 21.5 µg/ml, 2.5× DC = 53.8 µg/ml, 5× DC = 107.5 µg/l, 10× DC = 215 µg/ml; and (iii) alpha-cypermethrin: DC = 12.5 µg/ml, 2.5× DC = 31.3 µg/ml, 5× DC = 62.5 µg/ml, 10× DC = 125 µg/ml. Negative controls were bottles coated with acetone, the solvent used to dilute the test insecticides. Knockdown was recorded at 10-min intervals from the time of exposure until 1 h post-exposure, at which time mosquitoes were then transferred to holding cages. A small piece of cotton wool moistened with 10% sugar solution was provided to the mosquitoes during the recovery period after the 1-h exposure, until 24 h post-exposure [26]. Mortality was recorded at 24 h post-exposure. The strength of resistance, also called intensity, was characterised by comparing the mortality at 24 h for each of the insecticides at the various multiples of the diagnostic dose (Table 1). Resistance intensity was classified as low, moderate or high using a threshold of 90% for determining a resistant population at the diagnostic dose (1× DC) [27, 28]. The CDC bottle assays were used to determine resistance intensity as they allow for evaluation of customised multiple concentrations of an insecticide [24].

**Table 1:** CDC bottle assays for Chakanira and Sisewu villages

**Village**	**Insecticide**	**Dose**	**No. mosquitoes exposed**^**a**^	**Unadjusted % mortality**	**Adjusted % mortality**^**b**^
Chakanira	Control	0×	133	9.0	0.0
	Deltamethrin	1×	38	84.2	82.6
		2.5×	90	82.2	80.5
		5×	75	94.7	94.1
		10×	71	100.0	100.0
	Permethrin	1×	73	93.2	92.5
		2.5×	62	93.5	92.9
		5×	68	97.1	96.8
		10×	77	100.0	100.0
	Alpha-cypermethrin	1×	52	84.6	83.1
		2.5×	53	86.8	85.5
		5×	74	95.9	95.5
		10×	65	95.4	94.9
Sisewu^c^	Control	0×	25	8.0	0.0
	Deltamethrin	1×	21	90.5	89.6
		2.5×	25	92.0	91.3
		5×	38	94.7	94.3
		10×	17	100.0	100.0

^a^Low sample numbers could have affected these tests

^b^Adjusted mortality using Abbott’s formula as described by WHO [26]

^c^Due to limited mosquito numbers collected from Sisewu village, susceptibility tests were only carried out on one insecticide, deltamethrin

### WHO susceptibility tests

Wild-caught live female *An. funestus* mosquitoes were tested for phenotypic resistance to permethrin (0.75%), dichlorodiphenyltrichloroethane (DDT) (4%), bendiocarb (0.1%) and pirimiphos-methyl (0.25%) using standard WHO insecticide susceptibility assays [26]. The mosquitoes were exposed to papers treated with the respective insecticide for 1 h, knockdown was then assessed and the mosquitoes were transferred to holding tubes and provided with 10% sugar solution. Knockdown was assessed every 10 min from exposure to 1 h post-exposure, then mortality was recorded at 24 h post-exposure

###  Species identification by PCR

Following exposure the insecticides in the bioassay, a sample of the *An. funestus* population was subjected to species identification using PCR. Samples were analysed using standard operating procedures for identifying *An. funestus* group members [29, 30]. The PCR cycling conditions were: 94 °C for 2 min, 30 cycles at 94 °C for 30 s, 45 °C for 30 s, 72 °C for 40 s, with a final extension at 72 °C for 5 min.

### Data analysis

Minitab statistical software 20 was used to analyse data. Most data were summarised using descriptive statistics, including mosquito counts and mean mortalities. Mosquito mortality was calculated by dividing the number of dead/moribund mosquitoes over the total of mosquitoes exposed to a given insecticide/dose, then expressed as a percentage. Group means of mortality rates for different insecticides and doses were compared using analysis of variance (ANOVA). F-test* P*-values were reported from the ANOVA tests. The Tukey’s post-hoc test was then used for pairwise comparisons of group means at a confidence level of 95%, with the aim to look for differences in mean mortality rates among the insecticides and given doses, and determine by how much they differed.

### Results

A total of 1332 live wild *An. funestus* s.l. females were collected, of which 1153 came from Chakanira village and 179 from Sisewu village. These were used for the insecticide susceptibility tests (CDC bottle assays and WHO tube assays). A subsample of 400 (75 from Sisewu and 325 from Chakanira) was then selected for molecular identification. A small number of *An. gambiae* s.l. was also collected (1 specimen from Chakanira and 12 from Sisewu), but these were not processed further.

### CDC bottle assays

The results of the pyrethroid intensity tests for the two villages are summarised in Table 1, which shows the numbers of mosquitoes tested per insecticide concentration (dose), percent mortality rates and adjusted mortality rates using Abbott’s formula [26].

**Fig. 2:**
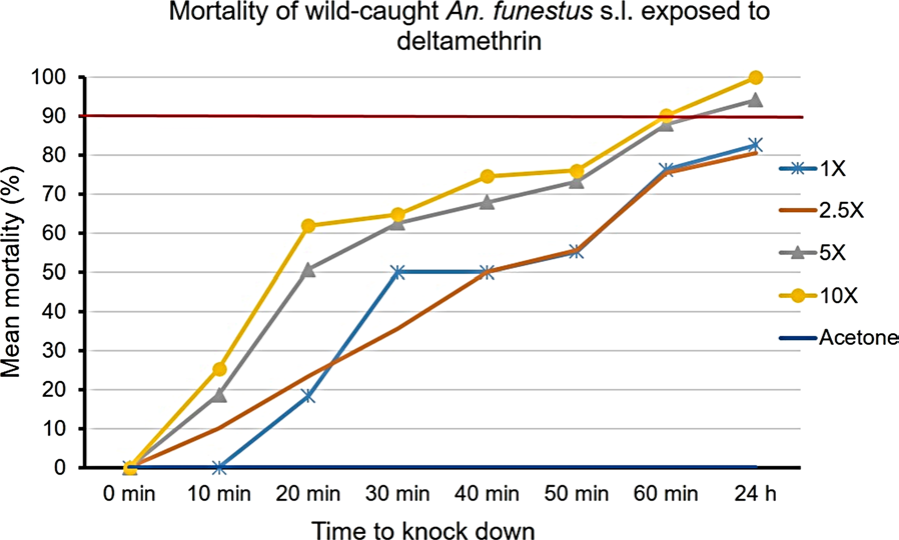
Knockdown time and 24-h mortality rates of wild-caught *Anopheles funestus* s.l., after exposure to various concentrations of deltamethrin. The horizontal red line represents the 90% mortality threshold for confirmation of resistance

**Fig. 3:**
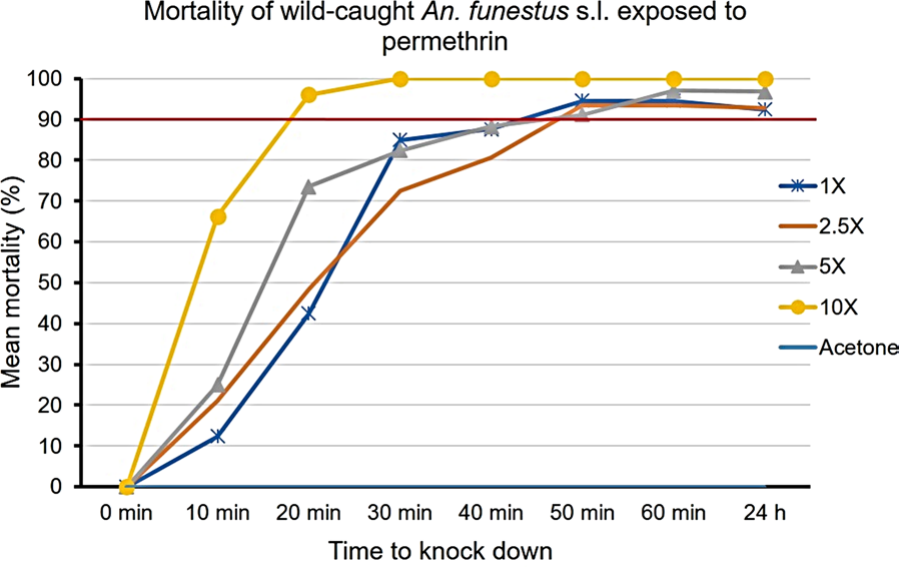
Knockdown time and 24-h mortality rates of wild-caught *An. funestus* s.l*.*, after exposure to various concentrations of permethrin. The horizontal red line represents the 90% mortality threshold for confirmation of resistance

**Fig. 4:**
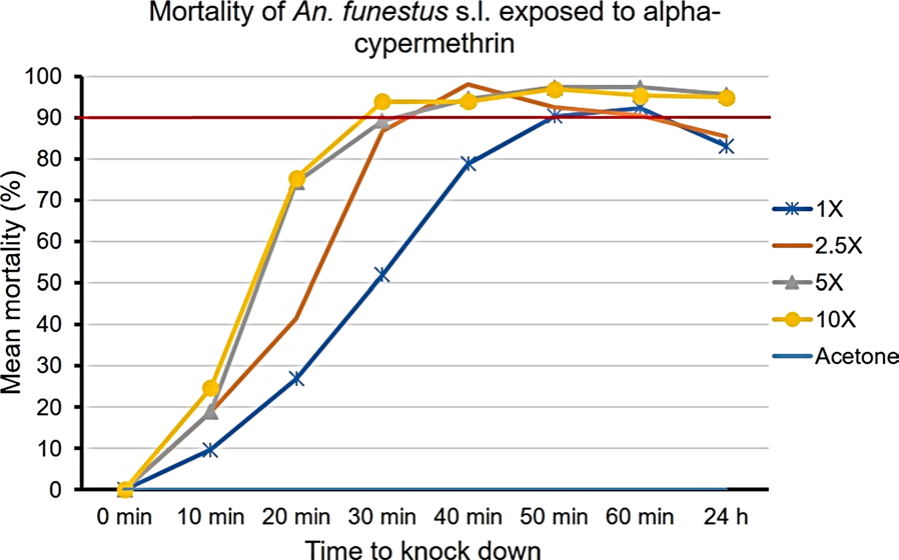
Knockdown time and 24-h mortality rates of wild-caught *An. funestus* s.l., after exposure to various concentrations of alpha-cypermethrin. The horizontal red line represents the 90% mortality threshold for confirmation of resistance

Figures 2, 3, 4 show the percentage knockdown of *An. funestus* s.l. from Chakanira village to the four concentrations of each of the three pyrethroids. Mortalities were adjusted using Abbott’s correction formula as per the WHO test procedures [26].

### Resistance intensity

**Fig. 5:**
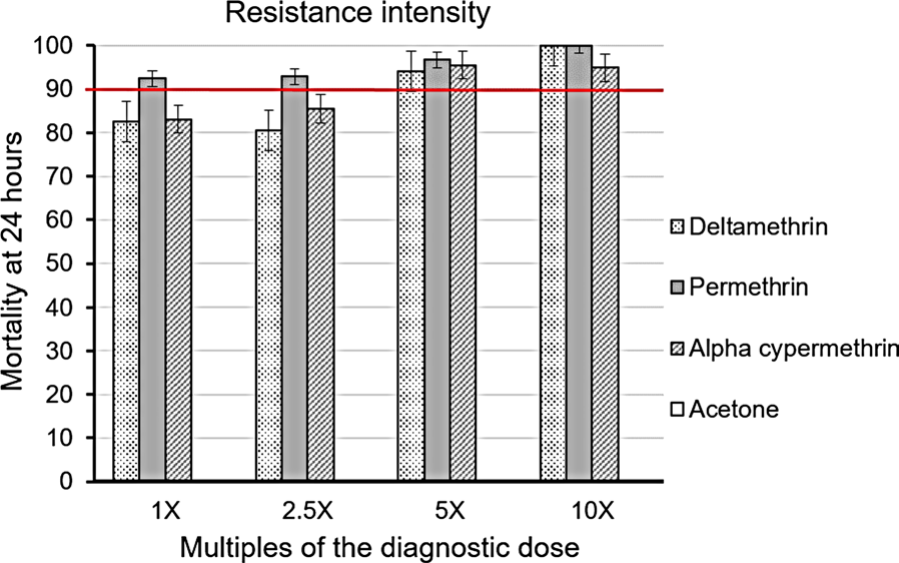
The response of wild *An. funestus* s.l. to different doses of the three pyrethroids tested. The horizontal red line at 90% represents the WHO threshold for a resistant population. Error bars represent standard error of mean mortality rates at 24 h post-exposure

Observed mortalities at the diagnostic dose (1× DC) were 82.6% (*n* = 38) for deltamethrin, 92.5% (*n* = 73) for permethrin and 83.1% (*n* = 52) for alpha-cypermethrin. These results confirm resistance to both deltamethrin and alpha-cypermethrin and suspected resistance to permethrin (Table 1; Fig. 5). For deltamethrin and alpha-cypermethrin, mortalities were still under the 90% threshold at 2.5× DC while they were in the range 90–98% at 5× DC in the Chakanira population [28].

**Fig. 6:**
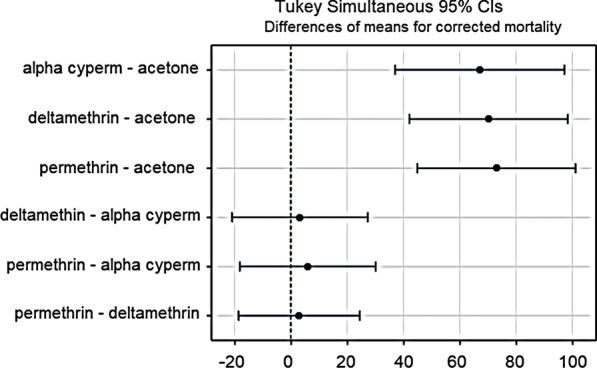
Pairwise comparisons of mean mortalities for deltamethrin, permethrin, alpha-cypermethrin and acetone negative control

**Fig. 7:**
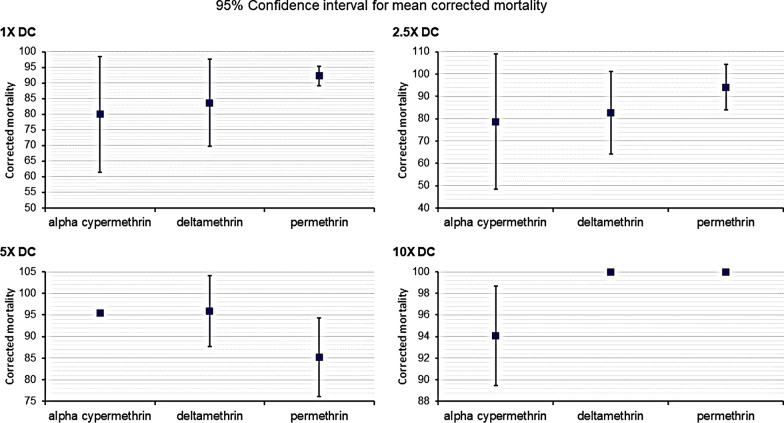
Interval plots for adjusted (corrected) mortality versus dose, for the three pyrethroid insecticides

For the given doses, ANOVA test results were as follows: 1× DC (ANOVA, *F* = 0.71, *df* = 2, *P* = 0.543); 2.5× DC (ANOVA, *F* = 0.49, *df* = 2, *P* = 0.636); 5× DC (ANOVA, *F* = 1.83, *df* = 2, *P* = 0.240; 10× DC (ANOVA, *F* = 4.77, *df* = 2, *P* = 0.087. Tukey’s post-hoc test was then used for pairwise comparisons of group means at a confidence level of 95% (Fig. 6). These test probabilities were all above the significance threshold (alpha = 0.05), which indicated that the test could not find an instance where the group mean for each insecticide at a given dose was statistically different from the others. In other words, the mean mortalities between the insecticides at each given dose belonged to the same population (were similar) and there was no evidence that any one of the insecticides performed better than the other at a given dose. This result was also indicated in the interval plots by the overlapping 95% confidence intervals of the means (Fig. 7).

### WHO susceptibility tests

From the total live collections, 408 specimens were used for WHO susceptibility tests against DDT, bendiocarb, pirimiphos methyl and permethrin (Table 2). Only a single WHO susceptibility test was done for Sisewu village on permethrin, due to low numbers of mosquitoes collected.

**Table 2:** WHO susceptibility tube assays

**Village**	**Insecticide**	**Dose**	**No. of mosquitoes exposed**^**a**^	**% Mortality**
Chakanira	Control	0×	29	0
	Dichlorodiphenyltrichloroethane (DDT)	1×	103	98.9
	Control	0×	27	0
	Bendiocarb	1×	93	33.8
	Pirimiphos-methyl	1×	103	100
Sisewu^b^	Control	0×	28	0.0
	Permethrin	1×	25	35.4

^a^Low sample numbers could have affected these tests

^b^Due to limited mosquito numbers collected from Sisewu village, susceptibility tests were only carried out on permethrin

### Molecular species identification

The molecular study for species identification on 400 wild females revealed that 98% (*n* = 392) were *An. funestus* s.s., followed by *Anopheles parensis* (1.5%, *n* = 6) and *Anopheles rivulorum* (0.5%, *n* = 2). While the majority of these specimens were from Chakanira village (81.2%, *n* = 325), 18.8% (*n* = 75) were from Sisewu village and were identified as follows: 72 *An. funestus* s.s., two *An. rivulorum* and one *An. parensis*.

## Discussion

The results of the present study highlight moderate to high resistance intensities in *An. funestus* from Chikwawa village against pyrethroid insecticides. Resistance to deltamethrin was confirmed in *Anopheles funestus* s.l. mosquitoes exposed to this insecticide at 1× DC, while in assays of these mosquitoes exposed to 2.5× DC and 5× DC, the mortality was < 98%. Susceptibility to deltamethrin was only restored when a 10× dose was used; hence, the classification of moderate intensity [28]. Similar results were observed for permethrin, with the only difference being that mortality at 1× DC was 92.5%, indicating possible resistance, which was confirmed when higher doses were used: at 2.5× and 5× DC, mortalities ranged from 90 to 98%. Susceptibility was also restored at 10× DC, similar to deltamethrin, indicating moderate resistance. Mortality to alpha-cypermethrin was < 90% at 1× DC and 2.5× DC, while it fell to 90–98% at 5× and 10× DC, indicative of a high intensity of resistance to this pyrethroid.

In addition to observing resistance against all three pyrethroids, we observed a low mortality (33.8%, *n* = 93) of *An. funestus* to the carbamate bendiocarb. However, intensity assays were not carried out in this study, and this observation warrants further study. Previous studies carried out in southern Malawi reported that *An. funestus* mortality to bendiocarb dropped from 60% in 2009 to 30.1% in 2014, then fell further to 19% in 2015 [16]. Unlike previous studies which reported moderate resistance or variable responses to DDT in the *An. funestus* s.s. population [13, 16], complete susceptibility to DDT (mortality 98.9%, *n* = 103) was observed in the current study. The present study also corroborates previous observations of full susceptibility to organophosphates, with an observed mortality of 100% (*n* = 103) to pirimiphos-methyl [11–14, 16].

One of the limitations of using wild mosquitoes is the possibility that they could have been exposed to various field conditions, including varying degrees of insecticide doses (e.g. on nets, wall surfaces or in agricultural fields), which potentially increases the risk of over-estimating resistance in the given mosquito population [26]. Older mosquitoes may tend to be weaker and hence more susceptible, risking an under-estimation of resistance [26]. However, this scenario more closely represents the actual conditions that any given intervention would encounter in the field; in addition, that wild-collected specimens are also the epidemiologically relevant cohort of vectors [23]. Changes in insecticide susceptibility in wild-caught vectors will thus more closely reflect changes in intervention efficacy [26, 31]. Another limitation of the current study is that we could not test many insecticides at all doses due to low sample numbers. However, even given the low numbers, we were still able to document the problem and its severity. Advanced molecular or biochemical tests for resistance mechanisms were performed as these were previously described in the study area by Riveron et al*.* [15].

The Malawi National Malaria Control Programme (NMCP) recently adopted an Integrated Vector Control Strategy (IVCS) 2020–2024 and the Insecticide Resistance Management Plan (IRMP) 2019–2022; these two programmes currently form the core of the malaria control programme in the country. For vector control, universal coverage of all at-risk populations with ITNs is the main approach together with complementary IRS in selected districts. In Malawi, ITNs are distributed via mass campaigns every 3 years, as well as through routine distribution in clinics to pregnant mothers and children under the age of 5 years, and IRS is implemented yearly in four districts [11, 32]. However, the implementation of IRS over the years has not been consistent in all the selected districts due to logistical and financial constraints and the rise in pyrethroid resistance. It was this increase in pyrethroid resistance that prompted the development of the strategic plan for monitoring and managing resistance (the IRMP) to counter the potential detrimental impact of resistance [11, 33]. The current study highlights the extent of the problem of resistance in Chikwawa village, warranting immediate action to safeguard progress in malaria control.

Although DDT is still effective against local mosquitoes and is recommended in the current vector control strategy, the potential use of this insecticide is hampered by concerns surrounding its use [34–37]. In 2006, the WHO made a call for the use of DDT for vector control and provided clear and strict guidelines which it adopted from the Stockholm Convention, stipulating how countries can incorporate DDT for malaria control in their malaria management programmes while strongly mitigating against any potential risks. Following the 2006 WHO recommendation for the use of DDT in highly endemic settings, Malawi announced that it would pilot a DDT IRS programme [36, 38]. Evidence shows that countries that continue both timely and correct use of DDT for IRS can reduce malaria transmission by up to 90% [39]. When South Africa reintroduced DDT spraying in 2000, they reduced the number of malaria cases and deaths drastically, managed to keep levels under control and, by 2012, set the goal to eliminate the disease entirely [40, 41]. The number of countries in Africa adopting DDT spraying for malaria control has risen, and since 2005, the list includes Malawi’s eastern and southern neighbour Mozambique, where *An. funestus* s.s*.* is a also key vector [42].

However, it has been argued that because Malawi has largely an agricultural economy, the benefits of using DDT for the fight against malaria may be outweighed by the potential detrimental effects on the agricultural industry and the economy at large [43]. This debate is also common in other African countries, and some studies have even suggested that the potential benefits of DDT for malaria vector control would be in the same order of magnitude as its detrimental effects on infant mortality [44]. Compounds such as clothianidin and deltamethrin/clothianidin combinations thus offer a good option for a rotation strategy with organophosphates in the IRS programme. The current vector control strategy also recommends PBO-nets and combination ITNs for areas with high levels of pyrethroid resistance [11, 33]. The present study supports and highlights the critical importance of this strategy as resistance intensity data have predictive value for making informed insecticide choices, to prevent compromised transmission control [27, 28, 31].

Clearly, a vector control strategy such as IRS that incorporates DDT and pirimiphos-methyl or other organophosphates may be a preferred means to manage the current situation of resistance. Such a strategy would serve as an interim solution as novel compounds are being developed or await registration and WHO approval and recommendation. Also, strategies that incorporate insecticides with new generation modes of action, or those that do not involve the use of insecticides at all, should complement ongoing interventions to avoid further risks that resistance may pose to the control programme. These findings also highlight the importance of making new generation ITNs (such as PBO-nets) readily and widely accessible to communities as they have demonstrated to have greater entomological and epidemiological impact than standard long-lasting insecticidal nets (LLINs) in settings of high insecticide resistance [31]. Such a multi-pronged approach will aid the country as it transitions towards elimination.

In this study, *An. funestus* s.s. was found to be the most abundant mosquito species, as also previously observed [12]. *Anopheles parensis* and *An. rivulorum* were also detected, and continuous surveillance and monitoring of these species needs to be routinely incorporated into control programmes, as these two species have been shown elsewhere to be contributing to transmission and also found biting humans indoors [45, 46]. Very few *An. gambiae* s.l. were collected (*n* = 13) in this study and no further investigations were done on these specimens.

## Conclusion

The level of resistance in Chikwawa village has grown over the years, and current intensity determined in the present study warrants an urgent solution. Our results highlight the importance of a resistance management strategy to cushion against the potential negative impact of resistance. The use of third-generation synergists or dual active ingredient ITNs, and pirimiphos-methyl- or DDT-based IRS would help to control the current resistance intensity in *An. funestus.* Such steps will prevent control failure and safeguard the control gains that have been achieved so far. More research needs to be done to further validate the intensity of resistance against carbamates.

## Data Availability

Datasets used for the analysis of findings of this study are available upon request via the authors’ email addresses.
